# Comparative analysis of hatcheries contribution to poor development of day-old chicks based on biological and immunological performance

**DOI:** 10.14202/vetworld.2019.1849-1857

**Published:** 2019-11-26

**Authors:** P. P. Yeboah, L. A. Konadu, J. A. Hamidu, E. A. Poku, D. Wakpal, P. Y. Kudaya, A. Dey, S. M. Siddiq

**Affiliations:** Department of Animal Science, Kwame Nkrumah University of Science and Technology, Kumasi, Ashanti, Ghana

**Keywords:** chick quality, day-old chicks, foreign chicks, local chicks, residual yolk sac

## Abstract

**Background and Aim:**

The quality of day-old chicks is a cornerstone to successful poultry production. Chicks with a poor quality start slowly in the field and may have high feed intake, poor growth rate, and poor feed conversion ratio. The current study aimed to assess chick quality challenges encountered from day-old chicks hatched in most commercial hatcheries in Ghana.

**Materials and Methods:**

A total of 300 day-old chicks each were obtained from commercial hatcheries in Ghana and Europe. The chicks were labeled as locally hatched broiler day-old chicks (LBDOC) and foreign hatched broiler day-old chicks (FBDOC), respectively. Chicks were reared and monitored from day old to 21 days post-hatch. Sample of chicks (n=25) from each hatchery was euthanized weekly at 1, 7, 14, and 21 days and blood samples collected for analysis. The parameters measured included physical, hematological, immunological, histological, and bacteriological characteristics. All data were analyzed by SAS Proc GLM at p<0.05.

**Results:**

The live weight of chicks was higher in FBDOC compared to LBDOC on the 1^st^ day. The chick length and shank length of FBDOC were longer than the LBDOC. The 7-day chick mortality was 6% in LBDOC as compared to 1.5% in FBDOC. The LBDOC also had a higher wet and dry residual yolk sac percentages as well as higher residual yolk sac fluid volume than the FBDOC. The rate of yolk sac disappearance of the FBDOC was higher than the LBDOC. More than half of the LBDOC had developed navel strings and leaky navel compared to FBDOC. The LBDOC recorded *Escherichia coli*, Proteus, *Streptococcus* spp., and Gram-negative bacteria in the residual yolk sac isolated through the 21 days while FBDOC recorded *E. coli*, Proteus, and Gram-negative bacteria. The intestinal villi count, lengths, width, and surface area were all not significantly different. The blood monocyte levels appeared higher in FBDOC than LBDOC, which give evidence of higher immunity in FBDOC than LBDOC.

**Conclusion:**

The results indicate a challenging situation in maintaining the quality of locally hatched broiler day-old-chicks compared to foreign hatched broiler-day-old-chicks. The study demonstrates that chick quality impact goes beyond the physical characteristics of chick weight and chick length, and the higher performance of FBDOC may be influenced by compliance with international hatchery standards and vaccination protocols.

## Introduction

The major constraint to the survival of the poultry industry is feed which accounts for about 60-75% of the entire production cost [1]. However, personal evaluation of this sector in Ghana indicates that most farmers often startup with a poor quality chick. This could have a huge impact on the profitability of their enterprise since the acquired chicks will be the first major cost incurred even before the feeding cost. Irrespective of the quality of feed fed once the start-up chicks are of poor quality, a farmer will likely run at a loss. Therefore, the quality of chicks can be considered the main cornerstone of the poultry production chain.

Chick quality assessment is very subjective. However, in Canada, it is widely accepted by the Ontario’s poultry industry that the following variables may indicate a high-quality chick: Vibrant, alert and active, well-hydrated, uniform, have low bacterial contamination, good level of maternal antibody, well-healed navel, and normal mortality <0.5% in the first 5 days. Poor-quality chicks tend to have higher feed consumption, lower feed conversion efficiency, increased total feed utilization, slowed growth rate, a lower final body weight of broilers, and increase laying age of layers resulting in increased cost of production in both broiler and layer breeders [2]. The main factor that decreases chick quality on hatch during incubation is temperature, especially late incubation temperature variation [3]. Higher incubation temperature increases heat stress, decreases yolk utilization, increases residual yolk sac weight, and increases the formation of belly buttons in chicks at the end of incubation [4]. These chicks will be classified as low-quality chicks and prone to disease infections [5]. Recent projections indicate that Ghanaian farmers import over 5 million day-old chicks annually as a result of increasing chick quality challenges from local hatcheries even though the cost of importing the chicks is twice or more the cost of local day-old chicks [6]. The situation has arisen from increased chick mortality and poor growth rate of local chicks eventually leading to lower profit [7]. Challenges such as high navel opening ranging from unclosed navel to more complicated issues such as big navel buttons and navel strings lead to yolk sac infections as a result of reduced immunity [8,9]. The large navel openings provide routes for disease-causing bacteria into the intestines, leading to high early chick mortality [8,10].

Since the current strains of broiler chickens have embryos recording higher embryonic heat production in overheated incubators [3,11], proper management of the incubation parameters including eggs for incubation as well as hygienic and sanitary conditions in breeder farmers and hatchery are imperative to increase the quality of day-old chicks [3,8,12].

The objectives of the current research were to examine the differences in chick quality between imported from Europe and locally hatched chicks, determine the rate of yolk sac disappearance and intestinal development of chicks from day old to 21 days post-hatch, and assess the immunity level of day-old chicks from hatcheries with different environmental conditions as measures of chick quality beyond the physical examination.

## Materials and Methods

### Ethical approval

In the absence of Animal Care Committee available at Kwame Nkrumah University of Science and Technology, Kumasi, Ghana, at the time of this research, the research was conducted and supervised by the team leader following experimental procedures approved by the University of Alberta Animal Care and Use Committee in accordance with the Canadian Council on Animal Care (2009) guidelines [13]. In addition to the above ethical clearance, we also made sure that the main objective to assess chicks coming from potentially good performing hatcheries in abroad was achieved by comparing with chicks hatched from local hatcheries to ascertain the inefficiencies in the local hatchery conditions. This comparative collection of chicks was also aimed at giving the research opportunity to understand both breeding farm and hatchery challenges associated with poor chick quality through both egg collection and chick hatching techniques. The research was, therefore, designed to avoid taking eggs but the end product (chicks), because taking only eggs and hatching in local hatcheries, would only tell one side of the story of poor chick quality and clearly demonstrate the challenges of the local hatchery where eggs would hatch. For this reason, chicks were imported from a foreign hatchery in Belgium.

### Location of study and experimental design

This research was carried out at the Department of Animal Science, Kwame Nkrumah University of Science and Technology, Kumasi. A total of 300 day-old chicks were obtained from foreign and local sources. This comprised 150 locally hatched broiler day-old chicks (LBDOC) of Ross 308 strain from a local hatchery in Ghana but supplied by Chicks and Chicken Co. Ltd., in Kumasi and another 150 foreign hatched broiler day-old chicks (FBDOC) of same strain and age, also obtained from Belgium through a local distributor called Reiss and Co. Ltd. and used as controls. It took the foreign day-old chicks about 24-36 h to reach the research site, but all handling procedures were made to reduce any stress on the birds. The study was repeated one more time with chicks from other unknown hatcheries but from the same countries. The second set of chicks of foreign origin was obtained from Belgium through a company called “Holland Akokor,” located in Ghana. Twenty-five birds in each group were dissected weekly for laboratory analysis.

### Rearing of birds

The day-old chicks were kept for 21 days (3 weeks). Birds were housed in battery cages to reduce disease infestation by direct contact with litter. All chicks were kept in prevailing ambient conditions with temperature ranging between 23 and 28°C during the period of the experiment. The lighting was provided by 2 of 100 W tungsten bulbs, which also provided heat during the day and night for brooding. The birds were kept in a raised battery cage about 2 m high above the floor with wire mesh flooring and the mesh covered with brown paper for the entire 3 weeks. Since the 3 weeks fell within brooding period, birds were not exposed to natural light. Black polythene was used to cover the cages and with the tungsten bulbs of 100 W this maintained heat for brooding. Vaccination schedules were followed as recommended by the Veterinary Service Department in Kumasi. The schedule included the first Gumboro vaccination on 7^th^ days, 14 days vaccination with HB1, and on 21 days intermediate Gumboro vaccination. All the vaccinations were administered through drinking water. Plain water was given after vaccine administration followed by an antibiotic with vitamins for 3 days to promote immune response and prevent disease outbreak while antibody production was underway. Coccidiostat was given each week to prevent an outbreak of coccidiosis. The chicks were fed *ad libitum*. The feeding and drinking troughs were washed and cleaned thoroughly daily to prevent infection from contamination through feed. The broiler mash had a crude protein content of 22% and energy level of 2900 kcal/kg.

### Physical observations during day old

On the 1^st^ day, all the birds were observed physically and the weight of chicks recorded a chemical balance. The total length of each chick was measured from the tip of the beak to the end of the middle toe. The chick was stretched sufficiently without causing harm or trauma to obtain an accurate measurement. The length of the shank was measured from the tip of the shank to the mid-portion between the feet using the Vernier caliper. The navel score was also assessed using the Tona score for the following parameters: Navel strings, navel buttons, opened navel, closed navel, leaky navel, and unhealed navel [14]. If the navel was completely closed and clean, it was scored 1 for navel quality. A score of 2 was given when the navel color was different from the chick’s skin color and had an opening <2 mm and chick navels with discoloration and a navel button more than 2 mm were scored 3 for navel quality. On a weekly basis, including on day old, 25 chicks (day-old chicks/birds) were randomly selected and weighed. The birds were euthanized, bled, and dissected to remove any residual yolk still remaining.

### Hematology and immunology parameters

After the physical examination, 25 birds from each treatment were euthanized by cervical dislocation and bled from the heart using a 2 ml syringe with a 23-gauge hypodermic needle into an EDTA tube and analyzed using a hematology auto-analyzer (Labcompare, South San Francisco, CA). Blood parameters analyzed included erythrocytes (red blood cells) and leukocyte (white blood cells). The white blood cells (differential count) were analyzed for monocyte percentage, neutrophil percentage, lymphocyte percentage, basophil percentage, and eosinophil percentage.

### Bacteriology

The residual yolk sac was removed after chicks were dissected from the abdomen and weighed, dried, and weighed again to determine the amount of fluid content in the yolk sac. A sample of the wet yolk sac was cultured to determine microbes present and the bacteria load. Culture media were prepared from commercially acquired agar base (blood agar base, MacConkey agar, and Salmonella-Shigella agar) and were prepared per manufacturer’s instruction (Bio-Rad, MacConkey Agar #3564154) and poured aseptically into sterile Petri dishes and allowed to cool down and solidify. The media plates were labeled accordingly and placed into an incubator at 37°C overnight to check for sterility and packed refrigerator.

When ready to use, the prepared agar media was removed from the fridge and dried in an incubator at 37°C for bacterial culturing. Culturing was done in a biosafety cabinet. A heat sterilized wire loop was used to take the matter from deep within the already prepared bacteria culture and streaks (lines made) on an agar plate and the plate incubated overnight at 37°C. The plates were examined the next day for bacteria growth. Smears were made from the bacteria colonies and stained using the Gram staining technique and further subjected to a biochemical reaction where Gram-positive organisms were expected to be stained as purplish while Gram-negative organisms were expected to be stained as pinkish [15]. Organisms isolated included *Escherichia coli, Streptococcus*, and Proteus.

### Histology of intestines

From the dissected bird, a piece of the intestine (ilium-jejunum junction) was cut and rinsed with sine solution to wash off intestinal content. It was fixed in 10% formaldehyde solution and after 48-72 h, the tissue was trimmed into smaller rings with a scalpel and fixed again in 10% formaldehyde solution to prevent the tissue from decomposition. The routine manual method was used for dehydration of the fixed tissues in various levels of alcohol, clearing in xylene, and embedding/impregnation in paraffin wax with corresponding time intervals [16]. Alcohol was used to wash off the formaldehyde solution from the tissue by gradually using the alcohol to dehydrate the tissue surface. The tissue was then transferred into xylene overnight to enable the effective clearing of the alcohol from the tissue to make the tissue receptive to infiltration media (paraffin) and also make it transparent or clear for examination. From xylene, the tissue was transferred into liquid paraffin wax and incubated at 56°C.

The melting or liquid paraffin wax was allowed to penetrate the tissue without causing any structural damage and converted the tissue to an easily manageable sample with a reasonable degree of elasticity. The tissues were blocked using rectangular metallic molds (Leica Mikrosysteme Vertrieb GmbH, Germany). The melted wax was poured into the molds and the tissue was gently pressed into the molten wax with the surface to be sectioned facing downward. The blocked tissue devoid of any air bubbles was allowed to cool gradually in the open air when it had solidified but still warm, immersed in cold water, to aid the cooling process. When the paraffin wax with embedded tissue was completely solidified, the block was removed from the mold and trimmed to the required size (30 mm in length by 20 mm in breadth). The rotary microtome (LabCE, American Society for Clinical Pathology) was used at this stage of section cutting. The cutting mechanism of the microtome was set at 10 μm to enable the acquisition of tissue sizes, thick enough to be observed under the microscope yet thin enough to be stained.

The mounted paraffin block was then secured in the holder and trimming was done to attain ribbon-like sections of the wax, containing cut sections of tissue. The ribbons from the trimmed layer were transferred into warm water in a water bath at 40°C for it to float and spread out. The floating ribbons were mounted unto an albumin greased slide and left on a slide rack to dry. Absolute alcohol was measured into two separate glass receptacles, alcohol of different concentrations (95%, 70%, and 50%) was measured into three glass containers, and distilled water was poured into a small plastic basin. All the reagents were labeled accordingly and a timer was set to the appropriate time and the separation of wax from the tissue was done. The dried sample was first placed in xylene for 2-3 min. Then, it was placed in the first absolute alcohol for 30 s and was again submerged in the second absolute alcohol for another 30 s. Then, it was placed in 95% alcohol for 30 s followed by 70% alcohol for 30 s and 50% alcohol for 30 s and finally washed thoroughly in distilled water. These steps were done to remove the wax from the tissue so that the tissue could be stained and observed under a microscope. The tissue sample was stained with hematoxylin and eosin [17].

The staining process included the mounting of a slide with tissue with a drop of Distyrene, Plasticizer, and Xylene (D.P.X.) mountant, covered with a coverslip. The D.P.X mountant was picked with a pipette and placed onto the slide and covered with a slide cover introduced at 40° and gently released into the slide. The dried slides were examined under the light microscope at 40× objective lens and a calibrated eyepiece. The villi of the intestines were counted and height measured by the count of the number of grid boxes that the villi occupied. To measure villi length, the number of grids occupied vertically was multiplied by 143 µm and the number of grids occupied horizontally was also multiplied by 143 µm and used as the measurement for the width. The surface area for the villi was calculated with the formula:

Surface area = πr√l^2^+r^2^

Where, I=length of villus and r=radius of villus.

### Statistical analysis

All the data were analyzed using the Proc GLM procedure of SAS 9.3 at p<0.05 [18]. Where significance was observed, the least square means were separated using the PDIFF procedure of SAS. The statistical model consisted of the fixed effect of the hatchery environment where chicks were hatched (locally hatched or foreign hatched) and the residual error term.

## Results

### Physical parameters of chick quality

**Figure-1:**
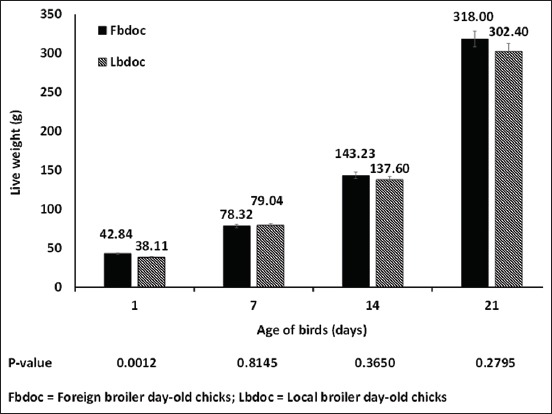
Live weight with respect to locally hatched and imported day-old chicks.

**Figure-2:**
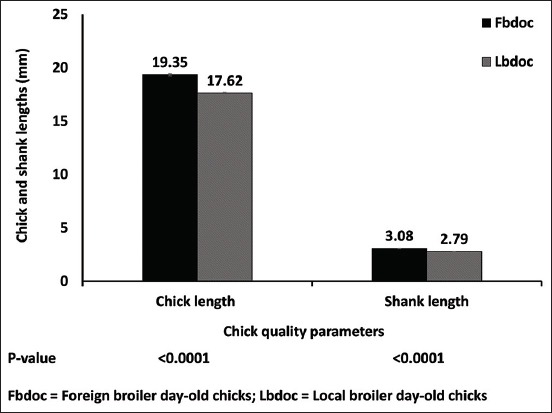
Chick length and shank length with respect to locally hatched and imported day-old chicks.

**Figure-3:**
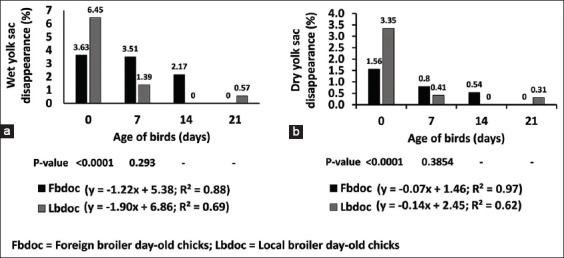
Rate of yolk sac disappearance with respect to locally hatched and imported day-old chicks.

**Figure-4:**
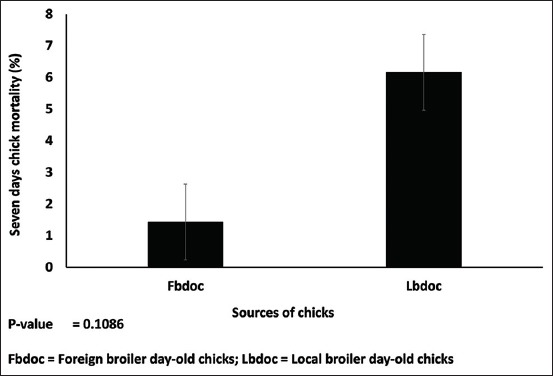
Mortality at 7^th^ days.

The live weight of the foreign chicks (42.84 g) was higher than that of the locally hatched (38.11 g) (p=0.012) (Figure-1). There was no statistical difference in the subsequent weights of the foreign and local chicks on day 7-21. The chicks were long (19.35 cm) for the FBDOC than LBDOC (17.62 cm) (Figure-2). Similarly, shank length for the foreign and local day-old chicks was 3.08 cm and 2.79 cm, respectively, which were statistically different from each other (Figure-2). There was a strong negative relationship between the wet and dry yolk sac disappearance and the age of the birds of the foreign broiler chicks as compared to the LBDOC (Figure-3). As illustrated in Figure-4, the LBDOC had high mortality on 7^th^ days post-hatch (6%) compared to the FBDOC (1.5%), contrary to expected high mortality of the FBDOC due to a potentially stressful condition on the FBDOC as a result of the 24-36 h of transportation from Belgium to Ghana. On the navel score for FBDOC, 66.1% had a clean and closed navel, 27.2% of chicks had navel opening <2 mm, 5.6% had a score of 3 indicating navel opening more than 2 mm, while 1.1% had navel strings. For the LBDOC, 27.0% had cleaned and closed navel, 30.7% had navel opening <2 mm, 12.9% had a navel opening more than 2 mm, 17.7% had navel strings, and 11.7% had leaky and unhealed navel (Table-1).

**Table-1:** The effects of source of day-old chicks on navel conditions according to the Pascal or Tona scores.

Navel score	Foreign broiler (%)	Local broiler (%)
1	66.1	27.0
2	27.2	30.7
3	5.6	12.9
4	1.1	17.7
5	0	11.7

1=Healed navel (closed and clean navel), 2=Discolored of navel with an opening of 2 mm or less, 3=Discolored navel with a navel button more than 2 mm, 4=Navel string, 5=Unhealed, open, and leaky navel

### Bacterial identification

**Figure-5:**
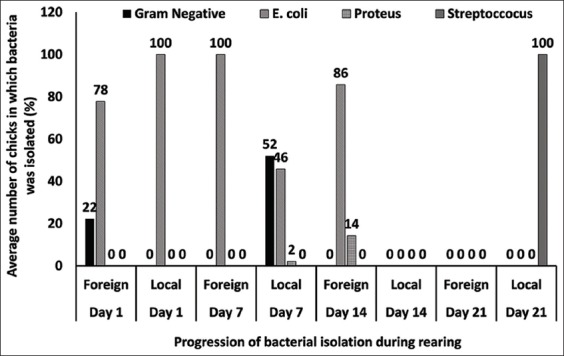
Bacteria identification.

*E. coli* was the only bacteria isolated from the LBDOC which formed 100% of the total bacteria isolated but was 78% in the foreign broilers and the remaining 22% were Gram-negative bacteria. On day 7, only *E. coli* was isolated from the foreign broiler day-old chicks (FBDOC), but *E. coli* and Gram-negative bacteria were isolated from the LBDOC. *E. coli* and Proteus were isolated from the FBDOC on day 14 with nothing in the LBDOC and on day 21, no bacteria were isolated from the foreign but *Streptococcus* spp. was isolated from the LBDOC (Figure-5).

### Immunology and hematology

From day 1 to day 21, the red blood cell count was consistently numerically higher in the FBDOC than the LBDOC (Table-2). The red blood cell count was high in the FBDOC compared to the LBDOC on day old and on day 7 (p<0.05). The white blood cell count of the FBDOC on day 1 was higher, but the level increased in the LBDOC on day 14 and day 21. Although the lymphocytes were higher only on day old in the FBDOC with the rest of the days being insignificant between the foreign and local chicks, the hemoglobin level followed a similar trend as the white blood cells. The monocyte count was higher in FBDOC compared to the LBDOC. However, on day 14, the level was higher in the LBDOC than the FBDOC and there were no differences on day 7 and day 21. There were no differences in the level of neutrophils throughout the 21 days. The FBDOC had numerically longer villi length, width, higher villi count, and larger surface area, but there were no statistical differences between these parameters with the LBDOC from day 1 to day 21 (Table-3).

**Table-2:** Effect of source of day-old chicks on blood immunological parameters over from day old to 21 days post-hatch.

Blood immunological parameters	Source	Day 0	Day 7	Day 14	Day 21
White blood count	Foreign	263.56	230.11	234.09	224.92
	Local	227.03	230.67	249.32	258.99
	SEM	3.29	3.93	5.02	4.69
	p-value	0.0001	0.92	0.0353	0.0001
Red blood count	Foreign	2.153	2.07	2.01	2.2
	Local	1.92	1.89	2.04	2.26
	SEM	0.08	0.05	2.03	0.06
	p-value	0.0387	0.0064	0.7004	0.527
Hemoglobin count	Foreign	7.54	7.75	6.63	7.87
	Local	5.74	7.87	8.34	9.76
	SEM	0.55	0.17	0.36	0.14
	p-value	0.0273	0.6259	0.0029	0.0001
Lymphocytes (%)	Foreign	85.83	93.25	96.17	92.26
	Local	95.54	94.1	96.59	96.31
	SEM	0.91	0.52	0.46	2.24
	p-value	0.0001	0.2535	0.5263	0.2039
Neutrophils (%)	Foreign	1.93	2.13	1.46	1.35
	Local	2.01	1.73	1.51	1.53
	SEM	0.0001	0.19	0.2	0.1
	p-value	0.7574	0.1337	0.869	0.1915
Monocytes (%)	Foreign	12.26	4.65	2.21	1.58
	Local	2.66	4.21	1.92	2.15
	SEM	0.83	0.38	0.3	0.97
	p-value	0.0001	0.4204	0.4912	0.0001

SEM=Standard error of mean

**Table-3:** Effect of source of day-old chicks on intestinal (ilium) villi characteristics from day old to 21 days post-hatch.

Intestine tissue	Source	Day 0	Day 7	Day 14	Day 21
Villi length (μm)	Foreign	330.49	374.48	378.95	425.70
	Local	303.88	311.24	365.20	370.70
	SEM	47.04	27.64	43.15	42.89
	p-value	0.7023	0.1158	0.8236	0.3736
Villi width (μm)	Foreign	209.73	239.53	232.38	240.90
	Local	168.03	264.13	210.65	223.30
	SEM	27.87	17.24	15.57	16.72
	p-value	0.3218	0.3209	0.3332	0.4640
Surface area (μm^2^)	Foreign	117,060.80	169,364.40	558,621.80	229,119.00
	Local	82,964.15	166,977.70	158,963.50	174,284.00
	SEM	22,095.77	34,038.43	335,025.60	67,698.52
	p-value	0.3076	0.9608	0.4070	0.5721
Villi count	Foreign	25	38	45	46
	Local	16	38	42	47
	SEM	3.64	3.72	4.05	5.29
	p-value	0.2222	0.9385	0.6862	0.6802

SEM=Standard error of mean

## Discussion

In defining chick quality, quantitative (weight or length) and qualitative assessment of day-old chicks are employed [12]. Factors such as the embryonic and/or day-old chick physiological parameters, chick weight, chick length, and chick physical look are important conditions needed to assess chick quality. Chick length is vital in acquiring the greatest uniformity and in predicting the growth performance of the chicks [19,20]. There is a positive relationship between chick length on hatch and chick weight on day 7. This confirms that embryo development can be expressed in terms of embryo length [19,21]. Chick length is also related to the yolk-free body mass and is also a good pointer of subsequent performance as well as final body weight [22]. The current study showed that the chick length was 19.35 cm for the FBDOC and 17.62 cm for the FBDOC, indicating the vast difference in chick quality.

There were similar differences in the chick weight and shank length which were higher in FBDOC compared to LBDOC. The ideal chick length for broilers is 22 cm [23]. Although the average lengths of both the FBDOC and LBDOC were below the standard length, there was a far difference comparing the standard to the local day-old chick average length. Various factors may contribute to the vast difference and may include the parent flock management, egg size, flock age, incubation practices, and post-hatch handling which may have affected the embryo development and reflected in the chick parameters. Chicks with the shortest chick length are found in hatcheries which have the highest 1^st^ week mortality problems and hatchery problems [19]. To remedy the problem, increase in chick length can be achieved when the age of the breeders is increased or a single-stage incubation system is employed to reduce metabolic developmental challenges [3,19].

Many researchers have illustrated that chick weight is the most common parameter usually used in the assessment of day-old chick quality. It has been proved that there is a direct relationship between a chick’s weight at hatch and its slaughter performance [24]. On a day old, a clear difference in chick weight was observed in the current study; however, its direct impact in final body weight is unknown because chicks are normally slaughtered from 42 to 56 days. The LBDOC had a lower body weight on day 1, indicating a need for careful grading and selection of eggs for hatching being cognizance of management procedures that optimize egg size for incubation [14].

The incidences of day-old chicks with subnormal conditions in the navel area can greatly impact the growth of chicks [12]. Chick quality parameters such as navel area abnormality are highly corrected with the amount of retracted yolk as observed in this study with respect to yolk sac disappearance. The higher rate of disappearance in the FBDOC may indicate better utilization of reserved yolk nutrients which could also be supported by long villi providing greater surface area for digestion. The disappearance also depended so much on the FBDOC having smaller residual yolk sac from day-old compared to LBDOC and, therefore, was used quickly in the FBDOC. The navel score is essential and reveals the quality and development of day-old chicks [10]. According to Bestman *et al*. [25], a chick of good quality should have a navel that is closed and completely healed with neither swelling nor buttons. The number of chicks with healed navel was about twice for FBDOC compared to LBDOC.

It is obvious from this research that the LBDOC could face challenges as to the number of chicks that can survive till the end of rearing should there be a disease outbreak. This is because navel abnormalities provided an opening for disease-causing bacteria into the intestines through the navel into the yolk sac and then the gut, since the small intestines are directly linked with yolk sac stalk, thus causing high early chick mortality [26]. These problematic navels such as unhealed navel are contaminated causing omphalitis or yolk sac syndrome that can lead to higher chick mortality [27]. This would lead to a lower final broiler body weight at 6 weeks post-hatch as well as higher chick mortality [27]. Moreover, chicks with big navel buttons have larger residual yolk sac and shorter intestinal villi and have delayed the absorption of yolk nutrients in the first 5 days post-hatch [10].

Acquiring poor-quality chicks mean that breeder or producer farms would have to regularly perform disinfection to kill infectious microorganisms. Omphalitis discussed earlier and also known as navel illness is the main cause of chick mortality and accounts for astronomical fiscal losses in the poultry industry [26]. This omphalitis is an inflammation of the chick navel and involves the yolk sac due to its close anatomical location resulting in about 5-10% post-hatch mortality in the 1^st^ week [28]. Chicks grow by absorbing nutrients from the yolk sac, thus indispensable to body growth. The yolk sac is infected by bacteria, especially *E. coli* at the time of egg collection for incubation and at hatching or right after hatching before the navel heals [29].

A higher antibody titer in hens indicates higher quantities of antibodies transferred to chicks and concurrently providing a long-lasting passive immunity [30]. This may be the reason for the high WBC count and monocyte percentage in the FBDOC observed in the current study. The obvious conclusion is that the local breeder hens need to be vaccinated more to provide higher antibodies to the LBDOC. Monocytes are precursors of macrophages [31] and higher levels provide high immunological competence to day-old chicks, and this may have to happen in FBDOC compared to LBDOC.

## Conclusion

Chick quality impact goes beyond the physical characteristics of chick weight and chick length which the poultry industry uses and the study of histopathological impacts is very important. The study concludes that locally hatched day of chicks compared favorably with the imported day-old chicks in terms of the absorptive capacity of the villi for growth and development. The bacteria identified and isolated were also similar and point to a comparable chick quality in these areas. Chick quality, however, appears lower in locally hatched day-old chicks in terms of navel score, immunological competence, residual yolk sac absorption rate, chick length, and shank length and 5-day mortality. Among the variety of factors influencing day-old chick quality, the paramount factors noted are breeder flock management, incubation practices, and vaccination among other post-hatch management practices. These may be the crucial causes of the differences observed in the local broiler and the foreign broiler and layer day-old chicks.

## Authors’ Contributions

PPY developed research protocols; PPY, LAK, EAP, DW, PYK, AD and SMS executed research experiments and data analysis. PPY, LAK, and DW drafted the manuscript. JAH designed the project and technical program, supervised the work, activities and data analysis, and revised and edited the manuscript. All authors read and approved the final manuscript.

## References

[1] Chiba L.J (2014). Section 12:Poultry Nutrition and Feeding. Animal Nutrition Handbook.

[2] Abera D, Abebe A, Begna F, Tarekegn A, Alewi M (2017). Growth performance, feasibility and carcass characteristics of Cobb 500 commercial broiler under small-scale production in Western Ethiopia. Asian J. Poult. Sci.

[3] Hamidu J.A, Torres C.A, Johnson M.L, Korver D.R (2018). Physiological response of broiler embryos to different incubator temperature profiles and maternal flock age during incubation. 1. Embryonic metabolism and day-old chick quality. Poult. Sci.

[4] Barri A, Oviedo-Rondón E.O, Wineland M.J, Small J, Cutchin H, McElroy A, Martin S (2009). Effect of incubation temperatures and chick transportation conditions on bone development and leg health. J. Appl. Poult. Res.

[5] Van de Ven L, Van Wagenberg A, Uitdehaag K, Koerkamp P.G, Kemp B, Van den Brand H (2012). Significance of chick quality score in broiler production. Animal.

[6] Djang-Fordjour H, Hamidu J.A, Adomako K (2017). Assessing incubation and performance deficiencies to boast broiler production. Am. Res. J. Agric.

[7] Hamidu J.A, Adomako K, Senanu J.M, Frimpong F.B, Bonsu F.R.K (2014). Comparative analysis of local and foreign hatched day-old chicks in Ghana. Ghana. J. Anim. Sci.

[8] Ulmer-Franco A, Cherian G, Quezada N, Fasenko G.M, McMullen L.M (2012). Hatching egg and newly hatched chick yolk sac total IgY content at 3 broiler breeder flock ages. Poult. Sci.

[9] Agbehadzi R.K, Hamidu J.A, Adomako K, Enu R (2019). Economic contribution of local hatchery performance in the poultry value chain in Ghana. Poult. Sci.

[10] Kawalilak L.T, Franco A.U, Fasenko G.M (2010). Impaired intestinal villi growth in broiler chicks with unhealed navels. Poult. Sci.

[11] Hamidu J.A, Fasenko G.M, Freddes J.J.R, O'Dea E.E, Ouellette C.A, Wineland M.J, Christensen V.L (2007). The effect of broiler breeder genetic strain and parent flock age on eggshell conductance and embryonic metabolism. Poult. Sci.

[12] Araújo I.C.S, Leandro N.S.M, Mesquita M.A, Café M.B, Mello H.H.C, Gonzales E (2016). Effect of incubator type and broiler breeder age on hatchability and chick quality. Braz. J. Poult. Sci.

[13] Canadian Council on Animal Care (2009). CCAC Guidelines on:The Care and Use of Farm Animals in Research, Teaching and Testing.

[14] Rajaravindra K.S, Rajkumar U, Rekha K, Niranjan M, Reddy B.L.N, Chatterjee R.N (2015). Evaluation of egg quality traits in a synthetic colored broiler female line. J. Appl. Anim. Res.

[15] Rad M, Esmailnejad K, Keleidar G.H (2003). Identification of gram-positive bacteria involved in yolk sac infection. Acta Vet. Scand.

[16] Hegazy R, Hegazy A (2015). Hegazy'simplified method of tissue processing (consuming time and chemicals). Ann. Int. Med. Dent. Res.

[17] Feldman A.T, Wolfe D, Day C (2014). Tissue processing and hematoxylin and eosin staining. Histopathology. Methods in Molecular Biology (Methods and Protocols).

[18] SAS Institute Inc (2012). SAS/STAT ®9.4 Procedures Guide.

[19] Hill D (2001). Chick length uniformity profiles as a field measurement of chick quality. Avian Poult. Biol. Rev.

[20] Wolanski N.J, Renema R.A, Robinson F.E, Carney V.L, Fanche B.L (2006). Relationship between chick conformation and quality measures with early growth traits in males of eight selected pure or commercial broiler breeder strains. Poult. Sci.

[21] Molenaar R, Reijrink I.A.M, Meijerhof R, Van den Brand H (2007). Relationship between Chick Length and Chick Weight at Hatch and Slaughter Weight and Breast Meat Yield in Broilers. Proceedings of 3^rd^Combined Workshop on Fundamental Physiology and Prenatal Development in Poultry, 5-10 October, Berlin, Germany.

[22] Molenaar R, Reijrink I.A.M, Meijerhof R, Van den Brand H (2008). Relationship between hatchling length and weight on later productive performance in broilers. Worlds Poult. Sci. J.

[23] Houghton H (2011). Disease Prevention in the Chick Embryo and Young Chick.

[24] Iqbal J, Mukhtar N, Rehman Z.U, Khan S.H, Ahmad T, Anjum M.S, Pasha R.H, Umar S (2017). Effects of egg weight on the egg quality, chick quality, and broiler performance at the later stages of production (week 60) in broiler breeders. J. Appl. Poult. Res.

[25] Bestman M, Ruis M, Heijmans J, Middelkoop K (2011). From Chick to Chicken. Chicken signals:practical guide for animal-oriented poultry. Second revised edition. Wageningen UR Livestock Research Postbus 65, 8200 AB Lelystad.

[26] Franco A.M.U (2011). Yolk Sac Infections in Broiler Chicks:Studies on *Escherichia coli* Chick Acquired Immunity and Barn Microbiology.

[27] Fasenko G.M, O'Dea E.E (2008). Evaluating broiler growth and mortality in chicks with minor navel conditions at hatching. Poult. Sci.

[28] Rahman M, Rahman A.Z, Islam M.S (2007). Bacterial diseases of poultry prevailing in Bangladesh. Res. J. Poult. Sci.

[29] Ganguly S, Praveen P.K (2016). Economically important poultry diseases of worldwide concern:A brief review. Int. J. Pharm. Biomed. Res.

[30] Sozcu A, Ipek A (2015). Quality assessment chicks from different hatcher temperatures with different scoring methods and prediction of broiler growth performance. J. Appl. Anim. Res.

[31] Gordon S, Plüddemann A (2017). Tissue macrophages:Heterogeneity and functions. BMC Biol.

